# Accelerated Researchers: Psychosocial Risks in Gendered Institutions in Academia

**DOI:** 10.3389/fpsyg.2018.01077

**Published:** 2018-07-19

**Authors:** Ester Conesa Carpintero, Ana M. González Ramos

**Affiliations:** Internet Interdisciplinary Institute, Open University of Catalonia, Barcelona, Spain

**Keywords:** gender, academia, time, well-being, precariousness, scientific careers

## Abstract

In recent decades, scientific institutions have undergone significant changes due to new managerialism and the application of excellence in research. This research model has given rise to tensions related to increasing pressures and working demands in a competitive international environment that accelerate the pace of academic life. In addition, precarious working conditions and job insecurity have affected academics’ lives and careers. Academic literature has already addressed these organizational changes and their impact on academics, however, few studies have focused on psychosocial risks related to time constraints, meritocratic pressures and career insecurity from a gender perspective. This analysis is relevant given the gendered distribution of responsibilities and the evidence of gender biases in academia that hinder the advancement of gender equality in scientific institutions, as the persistent lack of women at the top of research careers show. In this paper, we explore the psychosocial effects of the new organizational model of science characterized by accelerated time regimes and precarious working conditions from a gender perspective. We draw attention to gender-based discriminatory practices that may yield an accumulative effect on the well-being of women academics. We analyze 36 interviews from women and men researchers from five areas of knowledge in Spanish universities and research centers, following a ‘gendered institutions’ approach. The results highlight psychosocial risks for both men and women academics as a result of accelerated work organizations, intensified by uncertainty and hyper-competition due to lack of positions. The hegemonic male work model characterized by total availability confirms academia as a gendered institution, especially damaging women’s well-being and careers, as well as those of men committed to care responsibilities – challenging motherhood explanations – which may discourage them from the pursuit of gender equality. Our findings highlight discriminatory practices toward women academics which create psychological harm and feelings of being unwelcome, putting their career progression at risk. Lastly, we suggest a different model of work organization following the implementation of a culture based on an ‘ethics of care’ feminist approach.

## Introduction

New managerialist practices and the application of excellence and competitiveness in research institutions (Deem, 1998, 2001; Shore and Wright, 2000; Van den Brink and Benschop, 2012a) have increased working rhythms, accelerating the pace of the academic life (Ylijoki and Mäntylä, 2003; Gill, 2009, 2017; Walker, 2009; Vostal, 2015). This has impacted in researchers’ experiences, raising constraints in the academic practice and psychosocial risks, as some studies focused on academia have mentioned (Morley, 2005; Lynch, 2006; Sparkes, 2007; Menzies and Newson, 2008; Gill, 2009; Burrows, 2012; Leathwood and Read, 2013; Knights and Clarke, 2014; Mountz et al., 2015; Vostal, 2015). According to the European Agency for Safety and Health (Eurofound and EU-OSHA, 2014, p. 10) “‘psychosocial risks at work’ refers to the likelihood that certain aspects of work design and the organization and management of work, and their social contexts, may lead to negative physical, psychological and social outcomes.”

The audit culture that measure research performance through quantitative indicators, is identified as the origin of increasing stress and anxiety (Morley, 2005; Lynch, 2006; Burrows, 2012; Leathwood and Read, 2013; Knights and Clarke, 2014; Felt, 2017). Uncertainty over research careers and precariousness in the academic labor market also affect the working conditions shaping researchers’ career development and personal lives (Gill, 2009; Müller, 2014; Fochler et al., 2016; Bozzon et al., 2017; Heijstra et al., 2017). However, few studies are focused on psychosocial risks of time constraints and precariousness from a gender perspective (Acker and Armenti, 2004; Menzies and Newson, 2008; Gill, 2009; Mountz et al., 2015). Many studies report gender biases in academia – centered, among others, on the lack of recognition, old boys’ networks, gendered construction of scientific excellence and harassment (Fitzgerald et al., 1988; Rossiter, 1993; Wennerås and Wold, 1997; Steinpreis et al., 1999; Bagilhole and Goode, 2001; Gupta et al., 2004; Van den Brink and Benschop, 2012a; Jagsi et al., 2016) – which impede the advancement of women researchers and add an accumulative risk on their psychological well-being.

Our research explores psychosocial risks experienced by women and men academics emerging from accelerated time regimes and precariousness from a gender perspective. We also pay attention to specific gender-biased attitudes that may exacerbate psychosocial risks on women. The study focuses in Spanish academia, drawing on 36 interviews from five different research and academic institutions. We firstly present the literature review addressing: (a) the influence of acceleration and audit culture on the well-being and practices of researchers; (b) the impact of uncertainty and precarious working conditions on researchers; and (c) gender inequality in academia with a specific focus on time regimes. In the methodology section, we describe the design of the fieldwork and analysis. We adopt a content analysis methodology drawing on a *gendered institutions* approach (Acker, 1992). In the results, we show psychosocial risks from a gender perspective related to (a) time constraints due to increased work expectations, (b) lack of positions, labor precariousness and career uncertainty, and (c) invisible and specific discriminatory practices on gender basis. In the discussion, we underscore the main results and limitations of the study, and lastly, we explore policy implications and conclusions.

## Literature Review

### The Impact of Acceleration and Audit Culture

Scientific excellence has been “discussed primarily in terms of productivity” and measured by indicators as part of new managerialist audit practices in recent decades (Deem, 1998, 2001; Shore and Wright, 2000; Van den Brink and Benschop, 2012a, p. 508). These practices are based on “critiques of professional power” and used to boost outputs and to seek differentiation between individuals, institutions, and countries in a competitive international environment (Deem, 1998, p. 51, Deem, 2009). Indicators, that were originally conceived as sources of information, nowadays enact *academic value* – especially regarding journals impact factor – that guides crucial decision-making processes, such as funding achieved or career progression (Burrows, 2012).

Within these organizational changes, researchers’ demands and expectations have increased leading to an acceleration of academic working pace (Ylijoki and Mäntylä, 2003; Walker, 2009; Müller, 2014; Vostal, 2015). Vostal (2015, p. 298) points out tensions between increasing workload and unchanging temporal resources which “might have particularly misfortunate implications – for social environments, human relations, mental health and well-being.” In their study of time in academia, Ylijoki and Mäntylä (2003) find four temporal structures in conflict: schedule time (externally imposed timetables), timeless time (in-depth time needed for reading and writing), contracted time (referring to uncertainty and limited time of contracts) and personal time.

Some works mention audit culture as a source of stress and anxieties under constant self-monitoring and self-discipline (Morley, 2005; Lynch, 2006; Walker, 2009; Burrows, 2012; Knights and Clarke, 2014; Walker, 2014; Mountz et al., 2015). Morley (2005, p. 86) describes “stories of occupational stress, illness, alienation, fear, and resentment” among academics that highlight governance by numbers and rankings. On the one hand, the scarce time to perform research properly triggers feelings of vulnerability, failure, self-doubt, *imposter syndrome*, and occupational insecurity (Knights and Clarke, 2014). On the other, personal time for self-care, family or other interests, is “something that is lacking and constantly at risk of being excluded” which leads to academics’ burnout (Ylijoki and Mäntylä, 2003, p. 68).

Audit culture also affects the working environment as time constraints and output pressures give rise to individualistic strategies (Sparkes, 2007; Müller, 2014; O’Neill, 2014; Clarke and Knights, 2015). In this respect, some authors report that social relations are damaged and individual identities are *contaminated* by competitiveness, eroding friendly relationships and companionship in the workplace (Morley, 2005; Sparkes, 2007; Baker, 2010). Ostensibly, poor quality of work is also a side-effect related to the achievement of excellence as defined by quantitative indicators, which contrasts with a focus on a wider-ranging impact of the academic work – research, teaching and service – on society and the students (Lynch, 2006; Walker, 2009; Hartman and Darab, 2012; O’Neill, 2014). This also creates psychological tensions in academics such as feelings of frustration and a loss of meaning related to ethical and professional values (Knights and Clarke, 2014).

Grant culture or *projectification*, meaning the “organization of research through third-party funded projects” (Felt, 2017, p. 55), is becoming another major source of pressure to pursue research careers and to be promoted. Not only continuity depends on the funding achieved in many cases, but also professional identity and self-image become connected to the grants obtained (Knights and Clarke, 2014; Morley, 2016). Projectification also defines researchers’ strategies in predefined units of time: “[K]nowledge production must now be packaged in (generally) 3-year units, and publications are required during this time-span to demonstrate the worth of the investment” (Felt, 2017, p. 55). Research is structured in a standard and compressed way and different processes or unexpected events might not necessarily fit with the diversity of researchers’ experiences, which might cause stressful situations.

The promise of peer recognition sustains the work model of researchers in academia (Knights and Clarke, 2014), keeping them in a constant rat race for merits while dealing with the necessary time for self-care or the care for others. This creates conflicting feelings: “We [researchers] experience over-work, stress, guilt and anxiety as well as, if we are lucky, pride, relief and joy. We want to escape, but we are continually seduced by the potential pleasures on offer – either that and/or we simply need the job” (Leathwood and Read, 2013, p. 1172). However, expected rewards have a negative side as a result of rejection fear and unsuccessful aspirations, what is a highly frequent experience in academia – for instance, highly-ranked journals usually “reject 95% or more of submitted articles” (Gabriel, 2010, p. 763, cited in Knights and Clarke, 2014, p. 344).

### The Impact of Uncertainty and Precariousness

New managerial practices also attempt to “reduce public expenditure and impose tighter monitoring and auditing” (Deem, 1998, p. 51; Shore and Wright, 2000) that jointly with austerity measures have diminished working conditions. Permanent positions have decreased while new labor categories characterized by low salaries and instability have fragmented the labor force (Hey, 2001; Slaughter and Cantwell, 2012; Conesa and González, 2018). An increasing ratio of uncertainty and precariousness has extended in the form of short-term and/or part-time contracts (Steinthorsdóttir et al., 2016) which may aggravate anxiety. Following Gill (2017, p. 5), “[m]any are on zero hours contracts – or do not even have contracts – and often find themselves burdened with tutoring or grading responsibilities.”

Lack of permanent positions intensifies *hyper-competition* (Fochler et al., 2016) with a large number of academics forming a bottleneck (Conesa and González, 2018). This situation reinforces acceleration of working pace as researchers in early career stages and in non-tenure track positions struggle to increase their productivity in shorter periods of time with lower resources (Müller, 2014). In this competitive environment, working and living to anticipate and secure the future (Ylijoki, 2010; Müller, 2014) is fundamental since “[t]here is always someone who will work longer hours and produce even more ‘products’ to justify their position in the pecking order of the academy” (Walker, 2014, p. 62). Hyper-competition, therefore, hampers the rational utilization of work time and the adequate conditions to safeguard researchers’ well-being. Job precariousness and temporal constraints hinder researchers’ personal plans, such as having a family (Bozzon et al., 2017) because career stability arrives at later stages (Felt et al., 2017).

Budget cutbacks derived from the economic crisis have urged governments to push universities to apply for external funding, even though success rates are low due to wide competition (European University Association [EUA], 2015). For instance, the Horizon 2020 EU programs report a success rate of “approximately 14% in first 100 calls” (European University Association [EUA], 2015 p. 12). This pressure interferes in researchers’ work, particularly when they hold temporary contracts or they depend on grants to maintain their contracts, separating academics between winners and losers (Morley, 2016; Felt, 2017). Therefore, career progression relies on researchers who become responsible for their professional future (Gill, 2009; Leathwood and Read, 2013).

Some scholars have described this situation as an *affective economy*, indicating that hyper-competition and lack of career stability create emotional dependence on success (Müller, 2014; Fochler et al., 2016), which may lead to flawed scientific practices such as *salami-slicing*, text recycling or fraud (Lutz, 2012; Felt et al., 2017; Horbach and Halffman, 2017). Similarly, and according to Heijstra et al. (2017), fear of losing continuity makes academics in non-stable or precarious positions accept more time-consuming tasks, coined ironically *academic housework*. They usually accept the intensive work expecting a mid-term improvement in their position (Heijstra et al., 2017), perceiving that “a foot-in-the-door [is] a way of gaining a ‘proper job”’ (Gill, 2017, p. 5). Women are usually reported to sustain high rates of part-time work and fixed term contracts in academia (Van den Brink and Benschop, 2012a; European Commission, 2016, p. 102), as well as undertaking a high proportion of academic housework (Heijstra et al., 2017).

### Gender Inequality in Academia and the Impact of Academic Time Regimes

Although we would agree with the idea that not all academics experience accelerated academia as a constraining experience (Vostal, 2015), social factors such as gender may have a differential impact on women’s well-being. Many studies report inequality practices, revealing academia as a *gendered institution* (Acker, 1992; Van den Brink and Benschop, 2012a,b). The focus of these studies ranges from misrecognition and biased assessment procedures based on gender (i.e., deemed less competent, judged harder or judged on their physical appearance), to old boys’ networks and gendered scientific excellence (Rossiter, 1993; Wennerås and Wold, 1997; Steinpreis et al., 1999; Bagilhole and Goode, 2001; Gupta et al., 2004; Van den Brink and Benschop, 2012a,b) as well as sexual harassment (Fitzgerald et al., 1988; Jagsi et al., 2016), among others. Gendered practices governing academia indicate major pressures and difficulties for women overcoming psychological health risks.

Scientific excellence as assessed by quantitative rates and number of publications means that “time, and not quality, accounts for a large part of the appreciation,” which emerges as an unspoken rule that goes uncorrected for part-time researchers and those bearing more care responsibilities (Benschop and Brouns, 2003, p. 199; Van den Brink and Benschop, 2012a). As a gendered institution, academia reproduces the hegemonic male model of total time availability and devotion to work (Acker, 1992, 2006; Bailyn, 2003; Bleijenbergh et al., 2012; Bozzon et al., 2017). Care responsibilities are still understood as a women’s issue and rarely raised by men, leaving the model unquestioned (Van den Brink and Benschop, 2012b; Herschberg et al., 2014). Regarding the remaining gendered division of labor, some women are expressly unwilling to apply for promotion due to lacking the time and energy necessary for work-life balance (Baker, 2010). Although women currently working in the sciences are in no doubt about the importance of their professional careers and having more collaborative partners, there is still a gendered asymmetry of power in daily domestic and familiar responsibilities (González, 2014). This situation becomes especially onerous in international mobility periods, where women have to juggle complex decisions regarding their multiple roles (González and Vergés, 2013; González, 2014).

Although the topic of time and gender is often mentioned few studies have focused on women’s psychosocial risks, or the embodied effects related to time constraints and precariousness. Those that highlight these phenomena state that more research on this “too rarely discussed” topic is needed (Acker and Armenti, 2004; Gill, 2009; Mountz et al., 2015, p. 1236). High levels of health risks are reported, caused by sleep deprivation and fatigue – especially dealing with motherhood – and anxiety about future work, as the most common (Acker and Armenti, 2004). Gill (2009, p. 9) highlights that those women who want to have children might feel unable due to the intensification of demands that “make[s] it extremely difficult to manage” to do so, or job insecurity “that makes it too late.” Besides lack of sleep, Menzies and Newson (2008) report that women show higher rates of different indicators of stress compared to men. Due to their positions as ‘outsiders’ in academia, they have a greater pressure to present themselves as worthier, which may compel them to internalize to a large extent the precepts of high productivity expectations (Aisenberg and Harrington, 1988; Acker and Armenti, 2004). Embodied effects and affective states, such as overload, hurt, distress, shame, fear, isolation and guilt, are connected to fast regimes of time and quantitative metric-oriented careers in academia (Gill, 2009; Mountz et al., 2015).

## Methodology

### The Study

This study is developed within the framework of the GENERA project, which aims to compare scientific performance and academic cultures in different disciplines and research institutions in Spain from a gender perspective. We conducted 10 case studies based on a qualitative methodology including biographical interviews and document analysis of the recruitment policies, institutional web page content, and focus groups.

For this paper, we address psychosocial risks of women and men academics analyzing data gathered from interviews from five case studies located in different Spanish regions: three university departments and two research centres (see **Table 1**). They cover five fields of knowledge: humanities, architecture, telecommunications, environmental sciences, and biomedicine.

**Table 1:** Type of institution, field, and number of interviews.

Institution	Field	Number of interviews
Public University	Humanities	8 (4 men, 4 women)
Public University	Architecture	8 (4 men, 4 women)
Public University	Telecommunications Engineering	8 (4 men, 4 women)
Research Centre	Environmental Sciences	4 (2 men, 2 women)
Research Centre	Biomedicine	8 (4 men, 4 women)


We analyze 36 semi-structured biographical interviews (lasting from 60 to 180 min, audio-recorded and transcribed) conducted in 2015 and 2016. They are comprised of a balanced number of women and men at each stage of the research careers within these institutions (adjunct professors, junior and senior post-doc positions, associates, full professors, fellow researchers, senior researchers and leaders of research groups). All academics interviewed were full-time employees except four women adjunct professors. Their ages ranged from 28 to 67 years.

The biographical method based on personal interviews allows the researcher “to apprehend the prominent experiences from the life of a person and the definitions that person applies to that experiences,” therefore supposing an appropriate method to approach interviewees’ experience of psychosocial risks (Taylor and Bogdan, 1984/1992, p. 102). The subject matter of the interviews addresses professional and personal history, relevant moments in their careers, presence or absence of mentoring and institutional support, their experience on selection and promotion processes, scientific practices, time organization, future expectations and aspirations, and main obstacles experienced in their lives/careers.

A key informant from each institution put us in touch with the department director or a superior manager of the research centers who provided approval to undertake fieldwork – having been previously informed of the goals and characteristics of the study – and gave access to members of the institution, and to internal documents concerning hiring processes. The key informant also made initial contact with researchers, methodologically selected from a pool of candidates in correspondence with a theoretical sample and provided us their email addresses. We agreed to send a report outlining the main results and recommendations related to gender advancement and to receive their (voluntary) feedback, subsequently incorporated in the final report.

The participants were invited to take part via an email announcing the aims of the study, the methodology, the duration and procedure of the interviews (audio-recorded), and providing information regarding the privacy, confidentiality, and anonymity of the gathered data. A more detailed document was attached containing the name and funding program of the study, team members involved, abstract and main objective, methodology, our ethical commitment to the research, and communication of the final report prior to the publication of results. In this document we also provided information about the expected impact: the presentation and publication of results in scientific conferences and journals, and the dissemination of main results and good practices to policy bodies and to the general public and media. Following their positive response, we arranged a date for carrying out the interview and, immediately before starting the interview, we established verbal informed consent, stating the following: the objective of the study; that the interview would be audio recorded; that all personal data and information used would be anonymized and only accessible to the members of the study team; that they were free to ask any questions about the project at any moment, to stop the interview at any moment or to avoid replying to any of the questions for any reason. Verbal informed consent was obtained from all research participants regarding research participation.

Our commitment to safeguarding interviewee anonymity has resulted in the use of fictitious names and the erasure of any personal information that may identify them. Ethics approval was not required by the funding organization, national regulations, nor the university where the research was undertaken.

### The Analysis

We conducted a qualitative content analysis of the interviews. The analytical strategy is inspired by the method of constant comparisons in a spiraling process developed by Corbin and Strauss (1990/2015). Firstly, from an inductive process we highlighted the topics found in the interviews (codes) and, through several comprehensive readings, we detected commonalities (categorizations). Key questions around time, work intensification, precariousness, uncertainty, and discrimination emerged in connection with psychosocial risks and health issues and their possible relations to gender. Secondly, the quotations concerning previously mentioned key issues were constantly compared both within the interview discourse and between the interviews as a whole, examining pieces of data against each other to search for similarities and differences (Corbin and Strauss, 1990/2015). On one hand, this refines key questions into main categories (time regimes, working conditions, and discrimination practices) and, on the other, identifies variations and commonalities, leading the analysis to a more abstract level, following *theoretical comparisons* (Corbin and Strauss, 1990/2015). During a third phase, we applied consecutive comparisons contrasting interview discourses and main categories with the existing literature following the *gendered institutions* approach (Acker, 1990). Throughout this phase, we aimed to uncover novelties with respect to other studies, attaining in-depth and new knowledge. In a final phase, we reexamined our findings in order to assess the interpretation of data and select the most relevant quotations that connect the theoretical analysis with the fieldwork. In all of these phases we used analytical strategies, such as a special attention to language, expressed emotions, metaphors, different meanings of words, contrast examples and negative cases (Corbin and Strauss, 1990/2015).

Our gender analysis draws on the concept of *gendered institutions* developed by sociologist Acker (1992, p. 567): “The term “gendered institutions” [sic] means that gender is present in the processes, practices, images and ideologies, and distributions of power in the various sectors of social life,” referring to institutions such as the state, the economy, politics, or academia (Acker, 1992). Gender is embedded in organizational functioning, being that organizations are not gender neutral (Acker, 1990). Gendered processes are referred to by Acker (1992) more specifically as procedures that shape hierarchies, construct images and symbols, personal interactions based on *doing gender* and construction of the gendered self through ongoing accomplishment (see West and Zimmerman, 1987). According to Acker (1990, p. 568), “understanding how the appearance of gender neutrality is maintained in the face of overwhelming evidence of gendered structures is an important part of analyzing gendered institutions.”

## Results

A great variety of institutions make up the Spanish research and innovation ecosystem (universities, research centers, R&D enterprises, and other organizations) and they display different cultures regarding internationalization and competitiveness. In this study, time constraints are connected to high pressures surrounding research performance (i.e., publications and projects) and high teaching workloads in universities, although productivity expectations depend on departmental cultures and types of research center.

### Psychosocial Risks in Accelerated Male Time Regimes

Work organization and expectations in academia follow a time pattern that entails total availability related to high demands and research productivity. This feature of academic work is linked with hegemonic masculinity: “someone who gives total priority to work and has no outside interests and responsibilities” (Bailyn, 2003, p. 139; Acker, 2006; Bleijenbergh et al., 2012), that is, a white, middle-class and male breadwinner. In that context, personal time is neglected because of the centrality of work and the idea that a good and efficient academic should work long hours. This affects many women and men researchers following the same organizational and hegemonic masculine patterns. Therefore, those researchers with more responsibilities outside professional spheres – mainly women due to gendered division of work (Acker, 1992) – are highly exposed to psychosocial risks; although as we have found in the fieldwork, some men, committed with care responsibilities also endure similar difficulties.

#### Time Schedules and Obsession With Scientific Productivity

The academics interviewed devote more hours to work than the established in their labor contracts. Their schedule is determined as ‘extremely exhausting’ in the pursuit of merits and maximum productivity. Both women and men have interiorized this professional commitment which provokes psychosocial risks:

When I started the thesis, I worked from Monday to Sunday, 10 h a day, for 3 years and I burned out. It drained my energy, my strength and, finally, I decided not to work on weekends for my psychological well-being (…). You cannot work from Monday to Sunday for years without consequences. You realize that you don’t go out, you don’t have social life, I didn’t see my family (Miguel).

Despite his concerns about health risks, Miguel continues to overwork, as research performance is a requisite in the scientific career while time for family and social life can be relegated (Ylijoki and Mäntylä, 2003; Bleijenbergh et al., 2012). A culture of long hours is normalized to such an extent that not following this unwritten rule could mean, as Miguel states, that researchers do not cherish science sufficiently: “in science there is this culture that you have to suffer, otherwise [it seems] you don’t want it enough.”

Pressures related to the attainment of high citation impact and publication rates become an obsession for researchers given that these measurements *enact* academic recognition and value (Burrows, 2012). The *publish or perish* culture could easily entail abuse of working conditions, since academics may become caught up in institutional demands, misreading labor relations, which constitutes a threat to researchers’ well-being. In the quotation below, Mar explains how the high demands from her female boss exceed current legislation on working conditions:

My boss is a person who lives working 7 days a week, seven! And 12 months – or maybe 11 and a half – only working! I mean that she… her obsession is to publish articles, the greater the impact the better… and, if you have to do other things outside work it is simply not possible. You should leave whatever you want to do because you have to do this [research] right now! (…). One day I received maybe 10 or 15 emails from her saying ‘this is urgent’… whatever it was. Besides that, I received a text message, and if she had been able to come to my home, she would have come to. I knew what she was like this before and I accepted it! But also, I said to her that I didn’t want to live only for this [work]. And she accepted it too. I mean… (Mar).

Regarding international mobility, high pressure work situations intensify psychosocial risks because researchers lack support and networks (González and Vergés, 2013). Brenda felt under pressure in the United States, where her male boss did not allow her to have free time and holidays, compelling the entire research team to work all day long at the office. Brenda reports that she was living in a bubble in which only work existed (days meant an endless loop). Post-traumatic symptoms are evident even now whenever she receives her current boss’ calls. She highlights her difficulties in caring for her husband when he broke his leg in the US:

And then, he broke his leg, I was stressed because I didn’t have friends, everybody was at home, winters there [in the United States] last 7 months… I only ate junk food. It was the only thing that made me happy: to eat and smoke. My husband put on 17 kilos and I put on 11 kilos in a year. I ate a lot. It didn’t matter what you wore, you didn’t care about anything. You lose perspective… You have to go [to the office] the next day and deliver the results. If that night you can’t have dinner or you keep working until 3 am, so be it. Because it’s the only thing that matters in your life. And you are in this loop and it’s hard to get out. It’s difficult to stop. (…) And I’m still… my boss calls me and I jump and show up at the lab quickly. (…) It is still in my head. I feel frightened. I’m afraid of people saying ‘she is lazy’… (Brenda).

This high-pressure environment, abuse in power relations and lack of tools and support (she raises her concerns about her visa status considering the possibility of quitting the contract) eventually led to feelings of isolation and anxiety that produced a lack of self-care and an emotional dependence on giving results to her boss (Müller, 2014; Fochler et al., 2016).

#### Care and Professional Values in Accelerated Time Regimes

When interviewees talk about family responsibilities, women refer to tight schedules, scant sleeping hours, and high levels of exhaustion (Acker and Armenti, 2004; Mountz et al., 2015). Care work distribution with partners and the support of colleagues, especially in scheduling and in peer recognition, are crucial to maintain the necessary energy and motivation. Support from other relatives when it is not externalized – only mothers or female relatives are mentioned – is necessary to deal with high amounts of work or short-term mobility. Psychosocial risks appear more intense for women where partners are absent (single or divorced mothers), relatives are not close (or nonexistent) or home responsibility is recognized as unequally distributed. Flora, who leads two relevant international grants, displays high self-control regarding scheduling at work-life balance. She reports feelings of isolation owing to a lack of understanding from her colleagues who, she explains, want her to spend more time in the workplace; time with her daughters is paramount and care duties are unequally distributed between her and her partner. To solve this conflict, she has developed an exhausting time regime that she calls *being chronometered*, a timekeeping self-discipline that she implements to deal with work and family, avoiding any possible time wasting:

[If] You are the only one in a group of 12 people who is a mother or a father, it’s complicated… Let’s say that you feel different. You feel [like you are] in a different world, that… of course, you have chosen… but… you would also like to spend more time with them [her colleagues] instead of *being chronometered* all the time… Now, you see, I’m looking at the clock all the time, ‘I have 15 min left.’ It’s always like this, and it is very exhausting. But… could I do it differently? I don’t know… I could control my time less frequently but then I’m not with my daughters and that’s not a way to live. I feel responsible for them… it’s like a constant double responsibility (Flora).

Stress and depletion impact on researchers as a result of a work organization that outweighs spare time, family time and care time, since both family and care time are traditionally undervalued and unpaid work-time (Tronto, 1993; Torns, 2005). Even if women researchers strictly control time for work and family, they embody feelings of guilt due to a lack of time for caring, failing to accomplish other researchers’ expectations in a masculine work model, and failing to spend more quality time in each activity:

I always feel guilty about everything: the students, the colleagues… You know? I always leave [work] a bit early [to be with her daughters]. Then, I work every night but it’s like… Ok, you are putting your daughters to bed or giving them a bath, and you are thinking ‘Oh, I have to reply this email!,’ ‘Oh, I have to finish this!’

Flora places the responsibility for the situation as a whole on her choices and her own time management, assuming gendered clashing patterns: she deals with caring responsibilities while assuming the breadwinner role of a more-than-full-time work commitment. Tensions are intensified where there are high professional and family ethics and values that cannot be honored (Knights and Clarke, 2014). She does not question general work organization, nor does she call for a more reasonable and balanced time distribution, because she already experiences the lack of understanding of her colleagues without family responsibilities and the loneliness of being an outsider in a masculinized environment (Aisenberg and Harrington, 1988).

Few men raised similar concerns on family issues. Those who did expressed worry and distress about their productivity and career prospects because of difficulties in dealing with commitments in both spheres. Pablo explains that he is dealing with anxiety and describes himself as being a burden because he is no longer driven by high productivity: “Well, now, to break my back is more difficult. I mean, I have three children. Before I was in the lab every weekend and now I am only [there] exceptionally.”

A culture of excellence in science based on productivity (Van den Brink and Benschop, 2012a) creates harmful conditions that lead researchers to think they are not fast enough in terms of productivity, and as such that they are worthless. This condition displays an affective economy based on success dependency (Müller, 2014). Expressions such as ‘break my back’ show an extreme devotion to work and being burned out means *failure* in research performance norms. Mario explains that he is held back in his career when compared to those colleagues who have advanced faster than him. He cites that he is a picky person, working alone and methodically, and that family responsibilities compel him to spend more time with his wife and children than other colleagues do: “my family needs a lot from me.” Like many other academics in university, he values knowledge transmission and prefers devoting time to teaching (“[I] prioritize my students”) instead of research, and placing family before scientific productivity (Lynch, 2006; O’Neill, 2014). He represents a reversal of the traditional male model in academia and develops an alternative competitiveness based on an individual scheme that slows his publishing output.

Care responsibilities mentioned by men researchers is a novelty in the Spanish context, since it is a topic barely raised, as Herschberg et al. (2014) also note in the context of the Netherlands. Despite discourses of worry over career advancement, male frustration and anxiety seem to be more related to a desire to have time for family and to take on care responsibilities, while women’s appraisement of family is deeply interiorized and taken for granted. Male researchers are not outsiders within academia, whereas women attempt to engage in both spheres at the same level owing to an awareness that they need to demonstrate their value as workers (Aisenberg and Harrington, 1988).

### Psychosocial Risks and Precariousness: A Gendered Race for Scarce Resources

Austerity has weakened working conditions in academia. Among European countries, Spanish academia has been strongly affected by cutbacks (European University Association [EUA], 2015; Conesa and González, 2018). This creates a psychosocial impact on researchers connected to long-term precariousness, career prospects, family strategies and unwilling mobility. Lack of positions, especially tenured or tenure-track, is a common situation that increases hyper-competition, reinforcing an accelerated academy (Müller, 2014; Walker, 2014; Fochler et al., 2016). However, there are differences between universities and research centers.

#### Public Universities

Competitiveness and precariousness within universities are especially connected to a lack of positions rising from the freezing of replacement positions (Amoedo-Souto and Nogueira, 2013; Conesa and González, 2018). The result is a bottleneck situation in almost all public universities. This raises anxiety about the future as well as provoking the erosion of colleagues’ relationships, creating unfriendly working environments and emotional problems (Morley, 2005; Sparkes, 2007). Personal tensions lead to embodied effects such as somatization, internal fears, and loneliness. Marta’s words reflect this stressful environment where collegiality and well-being are at risk: “Because of these null replacement rates there are huge queues of people ready for promotion. And… it will be… a war! Come on!” (Marta). Tomás and his colleague, also a friend, had to deal with the situation of being offered the same position as lecturer. After receiving this offer, Tomás suffered abdominal pain as a result, on the one hand, to the need to compete against a friend, and on the other, to the fact that it represented an important step in a career offering very few opportunities for promotion or advancement: “When I came home, I had stomach ache… I mean… I had a knot in my stomach… I was sick… Well, I suppose that’s nerves…” (Tomás).

Long-term precariousness affects the careers and lives of researchers who become burned out and exhausted (Ylijoki and Mäntylä, 2003). Multiple demands combined with a lack of stability is a common formula for researchers expected to do more with less, absorbing their energy and motivation (Deem, 1998; Hey, 2001; Walker, 2009). In some universities there are long-term non-stable positions held by academics waiting on job vacancies for more than 10 years, contracted as temporary tenure-track associate professors or part-time, fixed-term adjunct professors with low salaries (Castillo and Moré, 2016).

Jorge explains that a long-term non-permanent position led him to burn out due to maintaining a precarious post for many years – a position that did not allow him to undertake research projects – all the while struggling with multiple demands and waiting for a position that never arrived. He had committed himself to maintaining a more managerial-based role as a foot-in-the-door (Gill, 2009) which led to personal and career setbacks. Dealing with the many quantified demands together with a precarious situation also affects the quality of teaching, clashing with professional values and bringing frustration (Lynch, 2006; Knights and Clarke, 2014):

All professors need to take on responsibility for the management of the university in order to understand how the university works, but it cannot be a priority because it makes you postpone research, it hinders your curriculum, and teaching also suffers. Students notice the lack of quality. You need to stop at some point because in the midst of so many demands, quality surveys, publications, stays abroad, excellence, teaching material… it is just impossible. (…)
For the last 2 or 3 years I have not had time to improve my teaching subjects: I have neither the head space nor the strength. I do not meet deadlines. (…) You end up burned out, profoundly burned out (Jorge).

Women in early careers hold adjunct professor positions (with one-semester or annual contracts and very low income), conducting *academic housework* and hoping their situation will provide the first step in their academic career, and are thus afraid of losing a very precarious position (Gill, 2009; Heijstra et al., 2017). This type of contract hinders career progression as it is designed for teaching support and stability is not guaranteed, a common situation in Spanish universities (Castillo and Moré, 2016). Sandra, in her forties, cannot advance in her research career despite a brilliant CV. Consequently, she conducts research in grueling working conditions:

This situation has been going on for the past 14 years and I’m tired because this position doesn’t allow you to apply for research projects, doesn’t allow you to… I mean, I renew the contract annually… I cannot create a research group, I cannot access funding, I cannot apply for European funding because my contract is very precarious and it is continuously renewed (Sandra).

Statistical reports from Spanish public universities (MECD, 2017) confirm Felt et al.’s (2017, p. 33) observations about the extension of the period during which scholars still count as junior, non-established academics. This situation generates feelings of helplessness and cynical responses: ‘Being stable when you are 45 years… It’s too much (…) I mean, mmmm, the future… (…) So… in my department, [laughs] this is the problem… The problem is that the Spanish university is a pile of shit and that’s all I can tell you’ (Cristina). This has consequences for both men and women’s life plans in terms of housing, family, and the economic security necessary for different life circumstances (Bozzon et al., 2017), creating feelings of insecurity, worry, anxiety, or rage.

#### Research Centers

In research centers job positions rely on grants and projects, and thus the culture of internationalization and hyper-competitiveness is pivotal. Early career researchers deal with anxieties surrounding job insecurity as they realize there are few available intermediate or permanent positions and large numbers of predoctoral or postdoctoral researchers, which leads Miguel to state: “a research career does not exist.” Brenda characterizes the workplace as hostile and unfriendly due to poor future prospects and high competitiveness:

(…) [T]he people that end up here are *very* competitive. There’s one position for 450 PIs [principal investigators]. We all know these statistics…Very, very, very, very competitive. And your best friends are never in your field because you’re fighting for the same grant, for the same money (Brenda).

Therefore, she refuses to become a principal investigator (PI), given that this position implies a total immersion in competitive and pressuring practices related to *projectification* and audit culture. Constant stress and limited resources become entangled in an affective economy (Müller, 2014) that leads researchers to feign being the best:

Would you like to be a PI?
No. No. I wouldn’t. The pressure they suffer… especially the young ones. Not those who have already built their fortress and live comfortably… The pressure they [junior PIs] are under to find money, the pressure to publish, the pressure they suffer to talk publicly, to pretend that you’re the best, otherwise they eat you. And this is related to your personality ehh… of…. of being the best of the best: ‘I don’t care about anything, and I never make mistakes.’

Brenda depicts the ideal researcher as a tough person, never making mistakes lest “they eat you.” Even if academic work is presented as neutral, hegemonic masculinity values characterize work and leadership styles where aggressiveness and hyper-competitiveness contrasts with a supportive, friendly and kinder style (Acker, 1992, 2006; Van den Brink and Benschop, 2012b; Morley, 2016). Brenda is also concerned about future uncertainty and lack of economic resources due to budget cutbacks. Austerity measures, limited time regimes, hegemonic masculine environments and a desire to have a family may push her to abandon academia and look for a job in a different sector. A self-protection response from psychosocial risks emerges from her words when comparing herself with another colleague working at the same institution:

‘(…) in my lab, there’s a guy who developed his career during the golden years of the leader [the boss] (…) Now he is 47 years-old. Now, the boss has no money. What is this guy going to do? I don’t want it to happen to me at 47 years old and with two children. I am still able to pack my bags and move, so I prefer to do it now.’

Gender, few grants available and accelerated time regimes intertwine in Flora’s decision, as she strives to overcome all these common obstacles. She defines academia as a rat-race, a pursuit of scarce resources (Müller, 2014), suffering from masculine hegemonic norms understood as neutral (Van den Brink and Benschop, 2012b). She is developing a brilliant career in a work environment where care work is worthless, which places her at a disadvantage (having to work faster in order to not fall behind):

A lot of women are in part-time work. In my case, I have done this Ph.D., I have went through everything for a goal [to be a scientist]… and I have a family! If I do not publish, if I do not have research projects, of course, I will lag behind the men, and if I am behind the men, I cannot win relevant grants and I won’t have other things [resources]. It was very clear to me: it is like this [to struggle bitterly for her career] or I have to start selling ice creams (Flora).

Academic aspirations taken in tandem with breadwinner and caring roles present genuine difficulties for women’s career progression. Pain and sacrifice represent a persistent state of affairs in academia in a gendered race for limited resources (Vázquez-Cupeiro and Elston, 2006; Ylijoki, 2010).

### Psychosocial Risks Due to Sexism and Gender Discriminatory Practices

We have already identified practices and patterns embedded in male organizations which support evidence of academia as a gendered institution (Acker, 1992; Van den Brink and Benschop, 2012a,b). In this section we outline specific but invisible gender discriminatory practices based on gendered personal interactions and power distribution (West and Zimmerman, 1987; Acker, 1992) that emerge as a source of psychosocial risks exacerbated by work organization in academia. These practices are often hidden by a lack of awareness on the part of men and women in academia and are related to lack of recognition, lack of authority and sexual harassment suffered by women, which lead to exclusionary effects such as feeling unwelcome. Only exceptionally, women express discomfort with discriminatory attitudes toward them, conveyed as anger, sadness, and frustration. We explore these psychosocial effects through the examination of evaluation processes, daily work, and particular events within the working environment.

The climate created in recruitment and selection processes strongly influences future actions and performance of researchers, encouraging (or discouraging) them to pursue their aspirations in academia. The evaluation process is stressful for candidates, such that disrespectful comments concerning personal life and doubts about professional competencies may cause harmful states. Sexism, deeply rooted in our society, appears in evaluation meetings, provoking discomfort and anxiety in early-career women. During a fellowship interview, Brenda was asked gender-biased questions from an evaluator who inquired about her husband’s plans – he also being a researcher – assuming it may affect her career:

I did the interview, one of them [evaluators] was lovely – there were two – but the other destroyed me. And, obviously, he was going to ask [uncomfortable] questions… but of course, these questions were already what I went through every day. ‘Oh, really? Why have you decided to come here? Do you think you are able to be a PI here? We don’t offer internal promotion here.’ And I replied: ‘Neither here nor any other place where I’ve been.’ ‘Is that so? And is your husband going to come along with you?’ (Brenda).

She related that her interview was difficult, and that the woman interviewed previously had left the room crying. Pressing women in the interviews appears as a legitimate strategy as it establishes the strength of character required to pursue a *challenging* career. Men, however, are not subjected to these kinds of questions imbued with gender stereotypes; firstly, the male model presupposes strong and secure candidates (Van den Brink and Benschop, 2012b), and secondly the breadwinner model takes for granted that men are in charge of family life while women are subordinated to male plans (Acker, 1992; Van den Brink and Benschop, 2012a,b; González, 2014).

Informal practices, such as their exclusion from decision-making and influential networks, prevent women’s progression in research careers (Bagilhole and Goode, 2001; Van den Brink and Benschop, 2012a). A senior female researcher in a male dominated institution explains that she had never been invited in decision-making meetings to pre-select future senior researchers which other senior, male colleagues attended. She states that this is not only a discriminatory practice but that it also has implications for diversity in the recruitment of researchers to the institution (i.e., not necessarily white male researchers). She feels angry and ignored since she is isolated from influential panels: “I mean, we [women] are not a flower jar for decoration. None of us!” (Tina).

Discrimination on a gendered basis is also manifested in the dismissal of women’s authority, misrecognition, verbal insults and even sexual harassment, which cause women discomfort and fear of losing their job. Inappropriate comments or insults are a hidden injury only mentioned in the corridors (Gill, 2009) creating toxic environments for women. Sonia explains that during a discussion about professional issues, her department director argued with her alluding her recent divorce: “He shouted that I was a nervous wreck because I was getting divorced. I felt very bad… (…) I thought that ‘a man does not receive this kind of comment!”’ (Sonia). Sonia expresses indignation and rage that he would use her personal situation as a means of dealing with a professional confrontation.

Offensive comments from other colleagues undervalue and misrecognize women’s research competence. She also relates another male colleague’s comment about her saying: “‘You have a rating of 19 on ResearchGate while I have 14. And I think this is because you are pretty. You have received a higher rating because of your photograph.”’ She explains her feeling regarding his comment: “I was stunned… Come on! Could it not be that they are interested in my publications?” She expresses indignation over the threat to her self-confidence and competence, considering his comments about her physical appearance as both inappropriate and sexist. This example illustrates that casual comments or even jokes between colleagues are still keeping women in a subordinated position.

Sonia spoke about sexual misconduct when she was a young student undertaking an internship in an automobile factory:

I had problems… just because I am a woman, I swear, because they treated me like a fool. I had two bosses in this company, one of them… he was good but the head of purchasing treated me as if I was silly! And, once… he… he touched me on my thigh (…) And I took his hand away and since that moment, there were bad vibes! Nothing else… between us, nothing else… Thereafter, I was unhappy in this job, the people… afterward, everything was bad (Sonia).

Such a situation came to generate negative feelings, disaffection in the workplace and finally the abandonment of her job. The “negative consequences for sexual non-cooperation” in rejecting *deliberate touching* was identified by Fitzgerald et al. (1988, p. 167) in academic settings. This signifies a double abuse: the unwanted touching behavior that leads women to feel uncomfortable (and carries with it the objectification of the female body at work, in addition to being treated as foolish), on top of the hostile environment following the incident (“bad vibes”) that threatens women’s well-being and constrains their career decisions (Connell, 2006).

## Discussion

By adopting a gender perspective, in this study we attempt to explore psychosocial risks that arise from an accelerated academy model (Vostal, 2015, 2016) embedded in precarious working conditions. We thus contribute evidence focused on time and gender regimes in academia and provide more in-depth knowledge about their psychosocial effects (Acker and Armenti, 2004; Menzies and Newson, 2008; Gill, 2009, 2017; Mountz et al., 2015).

We show academia as a gendered institution in which practices, images and values are defined by a male hegemonic norm understood as universal, neutral and disembodied (Acker, 1990, 1992, 2006; Connell, 2006; Mählck, 2012). Organizations are gendered, incorporating assumptions about gender in their performance and reproducing gender power relations (Acker, 1990).

Acceleration of academic working pace due to high and monitored expectations of scientific productivity, and reinforced by understaffing, increases workloads and corresponds to a work model that demands total time devotion and in which “family, community, and personal life are secondary” (Bleijenbergh et al., 2012, p. 23; Bozzon et al., 2017). Accelerated time regimes draw on excellence and new managerial practices that generate long working hours, relegating private lives and self-care linked to personal well-being. Obsession with accountability and publication rates affects both women and men, damaging their health, and potentially resulting in negative power relations that intensify psychosocial risks.

This analysis goes beyond the motherhood explanations that are often mentioned as a means of addressing the ‘issues faced by women’ in research (Bozzon et al., 2017). Although time for caring responsibilities affects women careers, this issue does not take into account the diversity of women researchers and their different responses as per their own values and goals. Instead, we propose an examination of gendered institutions and the ways in which scientific organization shape researchers’ careers and lives and especially hinder women’s careers. This analysis entails an understanding of a gendered distribution of roles as regards professional and care responsibilities. Support from actors close to women researchers (partners, family, colleagues, and superiors) are paramount, although they still do not prevent them from experiencing exhaustion and stress as a result of the accelerated academic pace. Moreover, this support is usually hard to come by, as it depends on many non-controllable factors, and is particularly problematic during international mobility (González and Vergés, 2013; González, 2014).

Those men who want to contribute equally in career and caring responsibilities – and who begin to dare to talk about it Herschberg et al. (2014) – also experience additional tensions in this accelerated and precarious labor framework, erected upon a universal and disembodied male identity as researchers. Academic work organization also penalizes non-traditional masculine identities, which may discourage more men from pursuing gender equality in the future. This finding reinforces our recommendations regarding the necessary changes in academic work organization with respect to researchers’ experiences, in order to prevent psychosocial risks and career disadvantages.

Psychosocial risks increase in parallel with job insecurity and precariousness. This yields hyper-competition (Fochler et al., 2016), erosion of collegiality, unfriendly environments, poor academic quality and burn out. Scarcity of positions intertwined with gender discrimination results in serious conditions for women who put up with an intense masculine work model jointly with caring responsibilities. Women make a great effort in the race for limited resources while trying to “manag[ing]e the unmanageable” (Gill, 2009, p. 11), taking on high levels of stress, discomfort and isolation.

Psychological harm is on the rise in gendered institutions, given sexism and discriminatory practices against women comprised of undermining, exclusion, isolation, objectification, mistreatment, and sexual misconduct. As Connell states, sexual harassment in gendered institutions impacts on women’s self-confidence in organizational settings (Acker, 1990, 1992; Connell, 2006, p. 838). Masculine power relations lead to feelings of being unwelcome that hinder their advancement within academic organizations (Fitzgerald et al., 1988; Jagsi et al., 2016).

### Limitations

As academic researchers, the authors of this study are aware of the risks of bias and preconception in the methodological process, especially in the analysis of interviewees’ discourses (Ylijoki and Mäntylä, 2003; Van den Brink and Benschop, 2012a). Taking into account that research is a situated human activity, we have tried to engage with *partial objectivity* (Haraway, 1988). We have been conscious of our positions while we have applied analytical distance through the constant comparison method and making connections with other research findings. This implies the revision of our own interpretations of academics’ discourses, and discussion between the co-authors of this paper as a means of examining different meanings and seeking out counterexamples to validate findings. Although our research is influenced by our own trajectory, we have consciously avoided dismissing those examples questioning our own prejudices, placing the participants’ words over our own experiences and understandings. Witnessing stress and worries about lack of time and self-care was the starting point for this research; some hypotheses were confirmed by the data from the fieldwork, whereas others were difficult to validate, such as, physical illnesses suffered by researchers that may remain hidden or neglected by interviewers’ responses. Only one women explicitly talked about sexual misconduct.

Despite difficulties in uncovering hidden symptoms, we found means of generating interviewees’ openness and trust that cast light on significant evidence. Moreover, some researchers explicitly showed willingness to articulate their experiences of stress, exhaustion, indignation and feelings of uselessness and exclusion.

### Policy Implications and Future Contributions

According to the evidence, the model of excellence based on quantitative indicators and high competitiveness needs to be addressed in academic organizations so as to promote well-being, quality in both research and teaching duties, and the inclusion of women in research institutions, particularly at senior stages and at decision-making levels.

Advancing in gender equality may require the application of an *ethics of care* feminist perspective that places care at the center of the political arena (Tronto, 1993; Carrasco, 2001; Mountz et al., 2015), and that counteracts a culture only based on (scientific) productivity and undervalues care work (such as ‘academic housework’). This perspective understands caring as a crucial activity “to maintain, continue and repair our ‘world’ so that we can live in it as well as possible” (Fisher and Tronto, 1991, p. 40; Tronto, 1993, p. 103), supporting ideas of interdependency and vulnerability linked to all beings. In this sense, it questions the disembodied hegemonic masculine model promoting an alternative gender-balanced organization of work and responsibilities.

This overhauls the underlying argument that takes for granted that women should adapt themselves to gendered organizations, as the development of work-life balance policies seem to support. These policies are ambiguous and fundamentally focused on women, avoiding tackling inequalities in multiple work, family, and societal spheres (Torns, 2005; Mescher et al., 2010). In the same vein, certain gender equality measures in academia such as mentoring, coaching, and quotas are only focused on “helping women to adjust to the male world,” instead of changing academic institutions (Van den Brink and Benschop, 2012b, p. 81).

Institutional changes should include an understanding of self-care and care for others as important aspects in the sustainability of personal and social life (Tronto, 1993; Carrasco, 2001), taking into account time availability as a powerful resource that needs to be equally distributed (Conesa, 2017). From an ethics of care feminist perspective, governments and policy-makers should *care about* the effects and consequences of new managerial practices and its accelerated time regimes, requiring *attentiveness*, *responsibility*, and *constant evaluation* – including *willingness to listen* to academics’ experiences for “managing the unmanageable” (Tronto, 1993; Gill, 2009, p. 11; Conesa, 2017). Changing academic institutions means, under an ethics of care feminist lens, to disrupt the exclusionary effects of gendered organizations.

In order to prevent psychosocial risks, we propose an extension of care culture, paying attention to work expectations and working time regimes, job security, healthy environments and respectful interactions which erode implicit and subtle biases. A culture based on the feminist ethics of care entails cooperation instead of competition, equal treatment and good working conditions, while promoting social justice and the encouragement of women’s and men’s advancement toward gender equality.

More concrete measures may include the assessment of quality through more qualitative than quantitative procedures (DORA, 2012; Hicks et al., 2015). As regards time regimes, an adaptation of schedule demands to more real and rational time resources (Ylijoki and Mäntylä, 2003; Vostal, 2015) is needed to take into account the sustainability of life, as well as a reversion of the long hours culture, that should be underpinned by labor rights. Other recommendations may entail a valorization of the different roles related to science and academic practice, and an understanding of the time needed to develop quality in teaching and research. Furthermore, less standardized and more flexible rules for projects and research outputs, in addition to an appreciation of diverse academic profiles and different types of epistemic cultures. Good working conditions should be guaranteed in order to prevent abuses of power driven by an obsession with productivity.

More research focused on the ethics of care feminist perspective to be applied in the academic context needs to be done. Psychosocial effects in the accelerated academy needs as well further contributions including an intersectional perspective that tackles other axes of inequality (Gill, 2009; Walker, 2009; Mountz et al., 2015).

## Conclusion

This paper addresses gender equality in academia by examining the psychosocial costs of accelerated working time regimes, job insecurity, and gender discriminatory practices brought about by excellence, audit culture and competition, adding a gender perspective. Going beyond explanations centered on motherhood, it tackles scientific organization via the *gendered institutions* approach (Acker, 1992), and suggests the application of an *ethics of care* feminist perspective (Tronto, 1993; Carrasco, 2001). Time constraints in academia penalize and exclude a diverse body of valuable researchers and approaches. This model, based on a hegemonic male norm, hinders women’s professional and personal lives, as well as men’s advancement toward gender equality.

## Author Contributions

EC designed the study for her doctoral thesis, conducted field work and analysis, and wrote the first drafts of the manuscript and successive revisions. AG principal investigator of the main project, conducted field work and analysis, and contributed to the successive revisions of the manuscript.

## Conflict of Interest Statement

The authors declare that the research was conducted in the absence of any commercial or financial relationships that could be construed as a potential conflict of interest.
